# Nutrient-mediated modulation of flowering time

**DOI:** 10.3389/fpls.2023.1101611

**Published:** 2023-01-19

**Authors:** Yuhang Zhang, Baohui Liu, Fanjiang Kong, Liyu Chen

**Affiliations:** Guangdong Key Laboratory of Plant Adaptation and Molecular Design, Guangzhou Key Laboratory of Crop Gene Editing, Innovative Center of Molecular Genetics and Evolution, School of Life Sciences, Guangzhou University, Guangzhou Higher Education Mega Center, Guangzhou, China

**Keywords:** nutrient, flowering time, nitrogen, phosphorus, potassium

## Abstract

Nutrition affects plant growth and development, including flowering. Flowering represents the transition from the vegetative period to the reproduction period and requires the consumption of nutrients. Moreover, nutrients (e.g., nitrate) act as signals that affect flowering. Regulation of flowering time is therefore intimately associated with both nutrient-use efficiency and crop yield. Here, we review current knowledge of the relationships between nutrients (primarily nitrogen, phosphorus, and potassium) and flowering, with the goal of deepening our understanding of how plant nutrition affects flowering.

## 1 Introduction

Plants must acquire at least 14 essential mineral elements for their growth, development, structure, physiology, and reproduction; these comprise both macronutrients and micronutrients (Epstein, 2005). Nutrient availability is tightly linked to flowering time, which requires resources for producing and sustaining sink tissues for reproduction (Sanagi et al., 2021). Deficiency or excess of nutrients results in a stress response that affects flowering time (Tanaka, 1986; Miyazaki et al., 2014; Sanagi et al., 2021). Flowering time control integrates external environmental factors (daylength, temperature, light, stress, and nutritional status) and endogenous signals from the plant itself (Kobayashi and Weigel, 2007; Ahmad et al., 2022; Khosa, 2022). The mechanisms by which several environmental factors (daylength, temperature, and stress) alter flowering time have been well characterized and reviewed (Cho et al., 2017; Fernández-Calleja et al., 2021; Freytes et al., 2021; Lin et al., 2021; Luo et al., 2021; Osnato et al., 2022; Preston and Fjellheim, 2022; Shi et al., 2022); however, there are fewer reports on how nutrients affect flowering time. In this review, we summarize what is known about the interactions between nutrients [primarily nitrogen (N), phosphorus (P), and potassium (K)] and flowering time. Genes previously reported to be involved in nutrient-mediated modulation of flowering time are summarized in **Table 1**.

**Table 1:** Genes involved in nutrient-mediated modulation of flowering time.

Species	Gene name	Nutrient	Effect	Reference
Arabidopsis	*NRT1.1*	Nitrogen	Floral promotion	(Guo et al., 2001; Gras et al., 2018)
	*FNR1*	Nitrogen	Floral promotion (1.47 mM N, 29.4 mM N), floral inhibition (117.6 mM N)	(Yuan et al., 2016)
	*CRY1*	Nitrogen	Floral promotion (1.47 mM N)	(Yuan et al., 2016)
	*miR172*	Nitrogen	Floral promotion	(Gras et al., 2018)
	*DELLA*	Nitrogen	Floral inhibition	(Gras et al., 2018)
	*SMZ/SNZ*	Nitrogen	Floral inhibition (3 mM KNO_3_)	(Gras et al., 2018)
	*SMZ/SNZ/TOE1/TOE2*	Nitrogen	Floral inhibition (1 mM KNO_3_, 3 mM KNO_3_)	(Gras et al., 2018)
	*FT*	Nitrogen	Floral promotion (1 mM KNO_3_, 3 mM KNO_3_)	(Gras et al., 2018)
	*SOC1*	Nitrogen	Floral promotion (1.25 mg N, 31.5 mg N)	(Olas et al., 2019)
	*NLP6*	Nitrogen	Floral promotion (31.5 mg N, short day)	(Olas et al., 2019)
	*NLP7*	Nitrogen	Floral promotion (31.5 mg N)	(Olas et al., 2019)
	*NLP6/NLP7*	Nitrogen	Floral promotion (31.5 mg N)	(Olas et al., 2019)
	*FBH4*	Nitrogen	Floral promotion (0.3 mM N, 3 mM N)	(Sanagi et al., 2021)
Rice	*NRT1.1a*	Nitrogen	Floral promotion	(Wang et al., 2018)
	*NHD1*	Nitrogen	Floral promotion (0.25 mM N, 2.5 mM N; 90, 180, and 360 kg N/ha)	(Zhang et al., 2021)
	*HD3a*	Nitrogen	Floral promotion (180 and 360 kg N/ha)	(Zhang et al., 2021)
	*DREB1c*	Nitrogen	Floral promotion	(Wei et al., 2022)
Arabidopsis	*NLA*	Phosphorus	Floral inhibition	(Kant et al., 2011)
	*PHF1*	Phosphorus	Floral promotion	(Kant et al., 2011)
	*miR399*	Phosphorus	Floral promotion	(Kim et al., 2011)
	*PHO2*	Phosphorus	Floral inhibition	(Kim et al., 2011)
Wheat	*PSTOL*	Phosphorus	Floral inhibition	(Milner et al., 2018)
Apple	*MYB2*	Phosphorus	Floral inhibition	(Yang et al., 2020)
Arabidopsis	*AKT2*	Potassium	Floral promotion	(Held et al., 2011)
	*CBL4*	Potassium	Floral promotion	(Held et al., 2011)
	*CIPK6*	Potassium	Floral promotion	(Held et al., 2011)
	*NaKR1*	Potassium	Floral promotion (long-day)	(Zhu et al., 2016)
	*SPL3*	Potassium	Floral inhibition	(Negishi et al., 2018)

## 2 Nutrients and flowering time

### 2.1 Nitrogen

Nitrogen (N) is the most important macronutrient for plant growth, needed for proper root morphology, shoot growth, stomatal opening, flowering, yield, and senescence (Bernier et al., 1993; Crawford, 1995; Marschner and Marschner, 1995; Leng et al., 2020; Sakuraba, 2022; Wei et al., 2022; Zinta et al., 2022). The influence of N on flowering time in Arabidopsis and rice can be visualized as a U-shaped trend (Lin and Tsay, 2017; Gras et al., 2018; Zhang et al., 2021), with both deficiency and sufficiency of N postponing flowering time.

The first evidence that nitrate is involved in the regulation of flowering time in Arabidopsis was obtained from genetic studies showing that *nia1nia2* mutants flower later than their wild-type controls (Tocquin et al., 2003; Seligman et al., 2008). Nitrate regulates the expression of flowering-related genes at the shoot apical meristem (SAM) to modulate flowering time; these genes include *SUPPRESSOR OF OVEREXPRESSION OF CONSTANS1* (*SOC1*), *CONSTANS* (*CO*), *FLOWERING LOCUS C* (*FLC*), *LEAFY* (*LFY*), *APETALA1* (*AP1*), and *FLOWERING LOCUS T* (*FT*) (Kant et al., 2011; Gras et al., 2018). NIN-LIKE PROTEIN6 (NLP6) and NLP7 function in the regulation of ${\text{NO}}_{3}^{-}$ signaling by binding to the promoter of *SOC1-LIKE3* (*SPL3*) and *SPL5* in the Arabidopsis SAM (Konishi and Yanagisawa, 2013; Guan et al., 2017; Olas et al., 2019). Nitrate greatly affects the vegetative growth of plants, so defining whether it has a direct influence on flowering is difficult (Hirel et al., 2007; Castro Marin et al., 2011). To separate the effects of nitrate on growth and flowering, a growth system using glutamine supplementation was established; low nitrate was still found to accelerate flowering in late-flowering Arabidopsis mutants with impaired photoperiod, temperature, GA, and autonomous flowering pathways (Castro Marin et al., 2011; Weber and Burow, 2018), suggesting that ${\text{NO}}_{3}^{-}$ regulates flowering time independently of the autonomous, light, and gibberellin pathways in Arabidopsis (Castro Marin et al., 2011). Moreover, delayed flowering time in *co*, *ft*, *fd* (*FLOWERING LOCUS D*), and *tsf* (*TWIN SISTER OF FT*) mutants as well as plants overexpressing *microRNA*156 (*miR156*) under low-N conditions proves that ${\text{NO}}_{3}^{-}$ -dependent flowering is indeed independent of age and photoperiod pathways (Gras et al., 2018; Olas et al., 2019). These observations indicate that ${\text{NO}}_{3}^{-}$ -dependent flowering pathways are dependent on the concentration and source of nitrate used (KNO_3_, NH_4_NO_3_, mixed, or supplemented with glutamine) (Gras et al., 2018; Fredes et al., 2019).

Protein phosphorylation is important for the transmission of information regarding N availability (Ho et al., 2009; Menz et al., 2016; Liu et al., 2017; Fredes et al., 2019; Liu et al., 2020). Notably, Sanagi et al. (2021) reported that the phosphorylation state of FLOWERING BHLH4 (FBH4) is altered by changes in N conditions, clarifying a link between N availability and the regulation of flowering. The kinase activity of SNF1-RELATED KINASE1 (SnRK1) is inhibited under low-N concentrations, resulting in a decrease in the phosphorylation of its direct target, FBH4 (Sanagi et al., 2021). This in turn promotes nuclear localization of FBH4, increasing the transcription of the flowering time genes *CO* and *FT* (Sanagi et al., 2021).

Delaying flowering by applying N fertilizers to crops is common in agricultural production, but the underlying molecular mechanism of this delay is largely unclear. High N delays flowering by improving transcription levels of SCHNARCHZAPFEN (SNZ) and SCHLAFMUTZE (SMZ), which directly bind to the promoter of *FT* (Mathieu et al., 2009; Gras et al., 2018). Mutants lacking ferredoxin–NADP oxidoreductase (FNR1) flower later than wild-type plants, and *fnr1* and *cryptochrome1* (*cry1*) mutants display insensitivity to different levels of N at flowering time (Yuan et al., 2016). FNR1 is inhibited at high N levels, leading to upregulation of CRY1 and degradation of FNR1 in the nucleus, thereby reducing transcript levels of central circadian clock genes [e.g., *TOC1* (*TIMING OF CAB EXPRESSION1*), *CCA1* (*CIRCADIAN CLOCK-ASSOCIATED1*), and *LHY* (*LATE ELONGATED HYPOCOTYL*)] and some flowering-output genes [e.g., *GIGANTEA* (*GI*) and *CO*] to disturb the flowering process (Pathak et al., 2013; Yuan et al., 2016). N signaling therefore alters the abundance of CRY1 protein and is also involved in the pathway of the central circadian clock, which regulates flowering.

Breeding programs often aim to develop cultivars that tolerate high N inputs without delayed flowering. OsNRT1.1A increases N utilization without delaying flowering, offering potential for the development of crops with the combined traits of early maturation and high yield (Wang et al., 2018). Rice, a short-day plant, has special flowering pathways besides the conserved flowering genes shared with long-day plants, such as Arabidopsis (Shoko et al., 2002; Doi et al., 2004). Arabidopsis and other upland plants prefer to absorb N as nitrate, while paddy rice prefers ammonium (Li et al., 2008); hence, these species use different mechanisms for regulating flowering time in response to N forms and concentration. (Zhang et al., 2021) showed that N-mediated heading date1 (Nhd1), which shares a high sequence similarity with CCA1 and LHY, increases the levels of florigen *Hd3a* to control flowering time but downregulates Fd-GOGAT for N assimilation in rice. Recent studies have shown that transcription of *OsDREB1C* (dehydration-responsive element binding) is induced by light and low N, suggesting a function in N-use efficiency and flowering time (Wei et al., 2022). Plants lacking this gene display delayed flowering under long-day conditions because OsDREB1C binds to the exons of *OsFTL1*, activating its transcription (Wei et al., 2022). As orthologs of *Nhd1* or *DREB* exist in other crops, the functions of Nhd1 and DREB in the regulation of flowering and N-use efficiency should be useful in other crops (Wei et al., 2022).

### 2.2 Phosphorus

Plants obtain phosphorus (P) mainly in the form of inorganic phosphate (Pi) (Marschner and Marschner, 1995). Low Pi availability generally delays flowering time in annual plants (Marschner and Marschner, 1995; Nord and Lynch, 2008); for example, the *nla* (*NITROGEN LIMITATION ADAPTATION*) mutant accumulates high levels of Pi and flowers significantly earlier than the wild type, while *phf1* (*PHOSPHATE TRANSPORTER TRAFFIC FACILITATOR1*) mutant with lower Pi accumulation flowers later than the wild type (Kant et al., 2011). *MiR399* and *PHOSPHATE2* (*PHO2*) are known to play a role in the maintenance of Pi homeostasis, and *miR399b*-overexpressing plants and *pho2* mutant accumulate high levels of Pi and exhibit early flowering phenotype *via* upregulation of *TSF* (Chiou et al., 2006; Kim et al., 2011). Moreover, the overexpression of *TaPSTOL* in transgenic wheat showed a significantly lower physiological P use efficiency and the P efficiency ratio than the wild type and were significantly later in flowering than the wild type, which revealing that P has a significant effect on flowering time (Milner et al., 2018). Transgenic expression of the Pi-responsive gene *MdMYB2* postpones flowering in Arabidopsis by decreasing the transcription of flowering genes (*CO*, *SOC1*, *LFY*, and *FT*) (Yang et al., 2020). No further data are available regarding the molecular mechanisms by which Pi availability affects flowering in crops such as rice, maize (*Zea mays*), and soybean (*Glycine max*); thus, further work is required to understand these processes in crop plants.

Phenological delay under low-Pi conditions is beneficial because it gives plants more time for P absorption (Nord and Lynch, 2008). By contrast, plants generally flower early to complete their life cycles more quickly under low-N conditions (Kant et al., 2011). Notably, crosstalk between N and P shows their accumulated influence on flowering (Kant et al., 2011; Yang et al., 2020). *MiR827* and *NLA* regulate Pi homeostasis in a N-dependent manner (Kant et al., 2011), and availability of both N and Pi affects flowering time by altering the expression of *FLC*, *CO*, *FT*, *LFY*, *AP1*, and some downstream genes of *miR156*–*SPL* (Kim et al., 2011; Vidal et al., 2014; Lei et al., 2016). The mechanisms by which N and P affect flowering time are worthy of further study, especially in crops.

### 2.3 Potassium

The concentration of potassium ions (K^+^) in plant cells can be as high as 100 mM, but the concentration in soil is only 100–1000 μM (Leigh and Jones, 1984; Wang and Wu, 2015; Wang et al., 2021). Absorption of K^+^ through roots requires K^+^ channels and transporters (Wang et al., 2021). In Arabidopsis, loss of *AKT2/3* (Arabidopsis K^+^ channel) changes the flowering time phenotype, implying that, similar to N and P, K^+^ availability also affects flowering time (Held et al., 2011); however, the molecular mechanisms involved are not clear. Additional studies report that *akt2*, *cbl4* (calcineurin B-like proteins), and *cipk6* exhibit analogous late flowering under short-day treatment (Held et al., 2011), indicating that regulation of ion channels involved in the flowering pathway and the activity of K^+^ channels are modulated *via* a Ca^2+^ sensor kinase (Held et al., 2011). (Negishi et al., 2018) found that mutant plants lacking *SODIUM POTASSIUM ROOT DEFECTIVE1* (*NaKR1*) over-accumulate Na^+^ and K^+^ and display late flowering. NaKR1 participates in the phloem transport of FT protein (Zhu et al., 2016) and increases the transcription level of *FT* under long-day treatment, dependent on K^+^ concentration. NaKR1 therefore regulates not only florigen production, but also transport (Negishi et al., 2018). It is thus clear that the *miR156–SPL* module might respond to N, P, and K, which deserves more in-depth study.

## 3 Perspective

Besides N, P, and K, there are at least 11 essential nutrients required by plants. Whether and how these nutrients affect flowering time require further exploration. (Yuan et al., 2016) suggested that FNR1 promotes flowering in response to N, and the level of *FNR1* is also induced by sufficient iron (Fe) and sulfur (S) supply. Therefore, Fe and S may also regulate plant flowering time through FNR1. Recent studies have mainly focused on the absorption, translocation, and reuse of nutrients (Verma et al., 2021; Wani et al., 2021; Johnson et al., 2022; Lambers, 2022; Liu et al., 2022a; Liu et al., 2022b; Podar and Maathuis, 2022; Prathap et al., 2022; Ren et al., 2022; Vélez-Bermúdez and Schmidt, 2022; Xie et al., 2022), meaning that the potential mechanisms underlying nutrient-regulated flowering remain largely unknown in plants, especially crops. UV stress, drought, salt, cold, and heat also alter flowering (Martínez et al., 2004; Cho et al., 2017; Ionescu et al., 2017; Shim and Jang, 2020; Preston and Fjellheim, 2022). Improving nutrient-use efficiency by coordinating flowering time is an effective way to increase crop yield; thus, the effects of interaction between flowering time and nutrients on crop yield are in need of more in-depth study.

## Author contributions

YZ and LC wrote this mini review, BL revised the mini review, FK and LC conceived the review. All authors contributed to the article and approved the submitted version.
